# *Mujeres Fuertes y Corazones Saludables*, a Culturally Tailored Physical Activity and Nutrition Program for Rural Latinas: Findings from a Pilot Study

**DOI:** 10.3390/ijerph16040630

**Published:** 2019-02-21

**Authors:** Rebecca A. Seguin, Cynthia K. Perry, Emma Solanki, Jean C. McCalmont, Judy P. Ward, Christie Jackson

**Affiliations:** 1 Division of Nutritional Sciences, Cornell University, 412 Savage Hall, Ithaca, NY 14853, USA; jpw243@cornell.edu; 2 School of Nursing, Oregon Health & Science University, 3455 SW US Veterans Hospital Rd, Portland, OR 97239, USA; perryci@ohsu.edu (C.K.P.); solankie@ohsu.edu (E.S.); jemccalmont@gmail.com (J.C.M.); 3 College of Public Health and Human Sciences, Oregon State University, 160 SW 26th St, Corvallis, OR 97331, USA; jacksoc2@oregonstate.edu

**Keywords:** health promotion, physical activity, nutrition, Latina

## Abstract

In the United States, Latino adults, compared with non-Hispanic white adults, are less likely to meet physical activity and dietary recommendations, and have higher rates of obesity. There is an urgent need for culturally adapted health promotion programs that meet the needs of the growing Latino population in the United States. We systematically adapted StrongWomen—Healthy Hearts, an evidence-based physical activity and nutrition program, for rural Latinas. This paper reports results from a pilot study of the adapted program. We used mixed methods to assess the feasibility and efficacy of the adapted program, *Mujeres Fuertes y Corazones Saludables*, in a nonprofit community organization serving rural Latinos. The intervention consisted of sixty-minute classes held twice weekly for 12 weeks and included 30 minutes of physical activity and 30 minutes of nutrition education. To assess efficacy, we used a one-group, pre–post design with overweight/obese, sedentary, middle-aged or older, Spanish-speaking rural Latinas (*n* = 15). Outcome measures included weight, height, body mass index (BMI), waist circumference, cardiorespiratory fitness, physical activity, dietary behavior, and self-efficacy for diet and physical activity. Process outcomes included attendance, end of class surveys, mid-program evaluation survey, and a post-program focus group. We calculated means and standard deviations, paproired *t*-tests, and Cohen’s D effect size. Qualitative data were analyzed using qualitative description. Significant changes pre- to post-program included weight (−1.5 kg; *p* = 0.009), BMI (−0.6; *p* = 0.005), waist circumference (−3.0 cm; *p* = 0.008), 6-minute walk test (69.7 m; *p* < 0.001), frequency of sugar-added drink consumption (−0.7 servings; *p* = 0.008), fruit and vegetable intake (1.3 servings; *p* = 0.035), and physical activity self-efficacy (0.9 points; *p* = 0.022). Participants found the program motivating and enjoyable, and on average participants attended 62% of classes and fidelity was maintained. This pilot study suggests that this culturally adapted physical activity and nutrition program for rural Latinas shows promise in improving physical activity, diet, and obesity.

## 1. Introduction

Poor diet and physical inactivity are key contributors to overweight and obesity, which are associated with premature mortality, hypertension, type 2 diabetes, cardiovascular disease, some cancers, low quality of life, depression and anxiety, body pain, and difficulty with physical functioning [1,2,3]. Only half of U.S. adults meet recommended levels of physical activity, and for all adult age groups fewer women meet physical activity guidelines than men [4]. While 53% of non-Hispanic U.S. adults meet the guidelines, only 43% of Latino adults do [4]. Similarly, Latino adults typically have poorer diets and higher rates of obesity than non-Hispanic whites [5].

This study adds to the growing body of knowledge surrounding the efficacy of interventions that are adapted for the Latino population. Since most research is conducted with dominant majority populations, and current research in Latino populations is not proportionate to Latino population growth, studies such as this one are essential for deepening our understanding of the cultural nuances within the Latino population [6]. Furthermore, most effective interventions have not been tested with minorities, and thus the external validity of them for these populations is not known.

Some lifestyle behavior interventions with Latinos have been successful in improving physical activity and other cardiovascular disease risk factors [7,8]; however, very few physical activity and nutrition interventions have included Latina farmworkers or rural Latinas, who may face additional challenges to attending health programs, such as geographic dispersion, lack of organization, sense of powerlessness, and transportation difficulties [9,10]. One pre–post study consisted of three one-hour nutrition classes for Latina migrant farmworker mothers; mothers increased nutrition knowledge and children decreased body mass index (BMI )percentiles [11]. Another intervention with immigrant farmworkers, consisting of 10 weekly sessions with *promotoras*, or community health workers, resulted in participants improving weight, waist circumference, fruit and vegetable intake, and physical activity [12]. A 12-month intervention with farmworkers with diabetes resulted in improvements in hemoglobin A1c (HbA1c), cholesterol, blood pressure, and social support [13]. Given the growing Latino population in the U.S. and the pronounced health disparities among Latinos, culturally adapted physical activity and nutrition programs offered in Spanish by skilled instructors are needed.

The original StrongWomen—Healthy Hearts intervention, tested in a randomized controlled trial in rural towns, was designed to address determinants of physical activity and healthy eating experienced by rural non-Hispanic white women [14]. Participants in the StrongWomen—Healthy Hearts program (53 intervention, 34 control) increased physical activity by 1637 steps/day (95% CI = 712, 2562), decreased saturated fat intake by 5.2 g/day (95% CI = −9.4, −1.0), decreased weight by 2.1 kg (95% CI = −3.2, −1.0), and increased self-efficacy for physical activity and dietary behaviors compared with participants in the control group [14]. The Cohen’s d effect size was large, with observed effect sizes ranging from 0.7 to 0.8 for time to walk two kilometers, steps per day by pedometer, and weight [14]. StrongWomen—Healthy Hearts is listed on the National Cancer Institute’s Research-Tested Intervention Programs website with scores (range 1–5) of 3.9 for research integrity, 4.0 for intervention impact, and 5.0 for dissemination capability [15].

In our work with rural Latina communities, rural Latinas expressed a desire for a culturally salient group-based physical activity and nutrition program for only women. We employed a systematic approach with a novel application of intervention mapping [16],to adapt a program that fit the goals and needs of rural Latinas; details of that process are described elsewhere [17]. Briefly, we first conducted a literature search to look for programs that matched the community goals and would require the least degree of adaptation. Based on that search we identified StrongWomen—Healthy Hearts. In order to adapt StrongWomen—Healthy Hearts for rural Latina participants, we created a community advisory board consisting of five Latinas representing various constituencies in the rural community (parent, high school employee, local nonprofit worker, social service agency worker, and community activist). During this year-long adaptation process, community advisory board members along with Dr. Perry and Dr. Seguin identified essential theoretical methods and program elements, adding or adapting elements to fit local context, reviewed and made changes to reflect the community and cultural context of the practical applications, intervention strategies, program curriculum, materials, and participant information [17], and planned the pilot study. Examples of adaptations made included a certified translator translating all participant materials into Spanish and having a bilingual class leader to increase accessibility; developing and integrating new participant information sheets to increase relevant health and nutrition knowledge on fruits and vegetables, sugar-sweetened beverages, meats and cheeses, and type 2 diabetes; incorporating additional participant materials to increase skills and strategies and reduce barriers associated with selecting lean protein sources, meal planning, shopping, meal preparation, and getting support from family and friends; replacing guidance on culturally unfamiliar foods and recipes with guidance on more culturally relevant foods and recipes; and increasing exercise options to include Latin dancing.

Because this is an adapted program, it is prudent to conduct a pilot study with a small number of participants to collect information on efficacy and feasibility before conducting a full-scale randomized controlled trial. This paper describes results of the process outcomes and behavioral and health outcomes from the pilot study of the adapted program, *Mujeres Fuertes y Corazones Saludables*.

## 2. Materials and Methods

### 2.1. Design

A pilot study was conducted using mixed methods with a one-group, pre–post design to assess feasibility and efficacy of the adapted program. In order to gather information on efficacy and feasibility, as well as areas for program refinement, both quantitative data (outcome measures) and qualitative data (open-ended questions on surveys and a focus group) were collected. Quantitative and qualitative data were analyzed separately. The qualitative data were used to provide context and understanding of the quantitative results and inform refinements to the program. This study was approved by the Oregon Health & Science University Institutional Review Board, number IRB00011637.

### 2.2. Sample

Participants were recruited from a town within a rural, agricultural county of Washington State with a Rural-Urban Commuting Area (RUCA) Code of 4.2, corresponding to a large rural area. The population in 2010 was 15,858 with 87.6% identifying as Hispanic, 48.8% female and 8.4% aged 65 and older. The median household income was U.S. $40,058, and 25% of households were living in poverty [18]. To be included in the study participants needed to be Spanish-speaking (mono or bilingual) Latinas, age 40 to 70, physically inactive (exercise two days or less per week), and live in the local community. Clinician authorization was required for any participant who answered “yes” to any question on the Physical Activity Readiness Questionnaire (PAR-Q). Exclusion criteria included having a health condition precluding moderate to vigorous physical activity. All participants completed an informed consent process in-person prior to being enrolled in the study.

Recruitment strategies included the use of bilingual flyers distributed in strategic locations in the community, local newspaper and radio advertisements, and church newsletters. Additionally, the community advisory board members were actively engaged in recruitment and recruited participants through informal networks, churches, schools, community events, family-oriented activities, heavily trafficked public places (e.g., grocery stores), and social media. They also assisted potential participants in obtaining clinician clearance. Since community advisory board members are trusted community members and knowledgeable of local cultural norms, they were approachable and were able to address potential barriers to participation including mistrust of research and non-local researchers. While this active one-to-one approach with the community advisory board members was resource-intensive, it yielded most of the participants. Based on input from the community advisory board, classes were held January to March in order to encourage farmworker participation due to their seasonal work schedules.

### 2.3. Outcome Measures

Measurements were taken at baseline and after completion of the program (at 12 weeks). The main outcomes for evaluating *Mujeres Fuertes y Corazones Saludables* were weight (BMI), waist circumference, cardiorespiratory fitness, physical activity, dietary behavior, and self-efficacy for diet and physical activity. The same measures from the randomized trial of the original program were used to allow for comparison across the studies. Measures that were not available in Spanish were translated by a certified translator. Height, weight, and waist circumference were measured by a family nurse practitioner. Aerobic capacity and endurance was measured with the six-minute walk test [19]; physical activity was measured using the International Physical Activity Questionnaire (IPAQ) [20]; and dietary behavior was assessed by the Food Intake Questionnaire [21] and the National Health and Nutrition Examination Survey (NHANES) Food Frequency Questionnaire [22]. Participants also completed a demographic questionnaire (e.g., age, marital status) and surveys regarding acculturation [23] and diet and physical activity self-efficacy [24]. Participants were compensated for completed data collection at each of the two data collection time points.

### 2.4. Process Measures

Class attendance was taken at each class as a measure of dose, accessibility, and acceptability of the program. Participants and class leaders completed a survey at the end of each class to assess fidelity to the program. Participants additionally completed a mid-program evaluation that asked about their likes/dislikes and suggested changes to the program. This evaluation survey allowed for participants to provide detailed, open-ended text responses. Four months after completion of the 12-week program a focus group was held to learn more about participants’ motivations and challenges in sustaining dietary and physical activity changes made during the program. Participants were compensated for focus group participation.

### 2.5. Intervention

The *Mujeres Fuertes y Corazones Saludables* intervention includes physical activity and nutrition education classes that meet twice-weekly for 12 weeks (total of 24 classes). The program is based upon Social Cognitive Theory, which focuses on how personal factors (e.g., self-efficacy) and environmental influences (e.g., social environment) interact. The program is specifically designed to enhance self-efficacy and social support. Class leaders employed team building strategies (e.g., greeting each person by name, setting group norms) to build group support, connectedness, and cohesion [25,26]. As part of the adaptation, we enhanced components aimed at learning how to garner support from family and friends. Each 60-minute class consisted of approximately 30 minutes of physical activity and 30 minutes of nutrition education Table 1 provides an overview of each class.

Over the course of the program approximately 50% of the class time was spent on physical activity in 30-minute sessions that consisted of 25 minute of moderate intensity exercise using an aerobic exercise or dance DVD that is progressive in nature, plus a 5-minute cool down at the end of each session. Aerobics and dance DVDs were chosen so that participants could follow the DVD (it was projected onto the wall) and the class leader could circulate and provide encouragement and feedback to individual participants during the exercise session. For each session, participants chose which DVD they wanted to use for that day, from a selection of aerobic exercise or dance DVDs. Class content included information on exercise intensity levels and choosing types of moderate intensity exercise. Participants were instructed to engage in moderate intensity exercise outside of class to build up to and reach the goal of 150 minutes of moderate intensity exercise each week (50 minutes being performed during the two class sessions each week). Types of moderate intensity exercise participants chose to perform outside of class varied, e.g., aerobics, walking.

Nutrition education included a variety of topics and activities focused on increasing nutrition and health knowledge and developing skills and abilities to support positive health behavior change. The nutrition component of the program was developed with the goal of shifting dietary patterns rather than focusing on specific nutrients according to the principles in the Dietary Approaches to Stop Hypertension (DASH) Eating Plan [27]. The food group and daily serving recommendation in the program followed the 2015–2020 Dietary Guidelines for Americans from the U.S. Departments of Agriculture and Health and Human Services [28] and introduced participants to principles of eating using HEART (“Heap on the vegetables and fruits, Emphasize the right fats, Accentuate the whole grains, Revere low- and nonfat dairy foods, and Target heart-healthy proteins”). Of the total time spent on nutrition education, those nutrition topics were discussed approximately 25% of the program time. Program time was also devoted to skill building and practice; this time included developing skills related to grocery shopping, meal planning, mealtime choices, and portion size as well as relieving stress and asking for social support from friends and family (25%). Class time was also devoted to self-monitoring skills and S.M.A.R.T goals (25%) as well as to group cohesion activities, food preparation and tasting, and assessments (25%).

Discussions on strategies to overcome social and environmental barriers to healthy lifestyle choices (e.g., support and sabotage from family/friends) are included in lessons. Participants set goals regarding exercise and healthy eating and were encouraged to keep exercise and food logs. To enhance participation and retention, class leaders maintained close contact with all participants. They called participants the day before each class to remind them of the class. Class leaders called participants who missed a class to check on them and using the spirit of motivational interviewing, assist them in developing a plan to overcome barriers, such as transportation challenges.

The classes were led by two bilingual staff members from a local nonprofit that serves rural Latinos. The instructors are well known in the community and are experienced community health educators. Similar to *promotoras*, who often lead effective behavior change interventions in Latino communities [29], the community health educators for the study had comprehensive training to lead the program and were selected due to their qualifications, as well as trust among community members. Leaders completed training on the original StrongWomen—Healthy Hearts curriculum through an online platform and participated in a supplemental training that included an overview of the adaptations and additional information on leadership skills and motivational interviewing. Throughout the course of the program, the class leaders and one of the lead researchers met weekly via phone to discuss questions, concerns, or issues and ways to address them.

### 2.6. Analysis

We examined descriptive statistics for baseline demographics. For outcome variables, we calculated means and standard deviations; paired t-tests; and Cohen’s D effect size. All tests were two-sided. Statistical significance was set at *p* < 0.05. For the process evaluation, we calculated descriptive statistics for the survey results. The focus group was held in Spanish. It was audio recorded, transcribed verbatim, and translated into English. The transcription and translation were checked for accuracy by a researcher proficient in Spanish. The transcript was analyzed using qualitative descriptive analysis, which is less interpretive than other qualitative approaches (e.g., grounded theory) providing straightforward description [30,31]. Our goal was to understand quantitative results and inform program refinements. Two researchers coded the transcript line by line independently and then met to compare codes, reach agreement, and develop categories and themes. Responses from the open-ended survey questions were reviewed to additionally inform program refinements.

## 3. Results

Fifteen women enrolled in the study and provided baseline data; 11 provided complete outcome assessment data (Table 2). Four participants dropped out within the first half of the program and although we contacted them about post-program data collection, they opted not to return for this data collection. No significant pre–post differences were found for income or employment status; these were examined due to known seasonal occupation variations among this population where farm work is common. Participants experienced pre- to post-program changes in weight (−1.5 kg; *p* = 0.009), BMI (−0.6 units; *p* = 0.005), waist circumference (−3.0 cm; *p* = 0.008), six-minute walk test (69.7 m; *p* < 0.001), daily frequency of sugar-added drinks (−0.7 servings; *p* = 0.008), fruit and vegetables consumed yesterday (1.3 servings; *p* = 0.035), and physical activity self-efficacy (0.9 points; *p* =0.022) (Table 3).

On average participants attended 62% of the classes. Barriers to attendance included weather and road conditions (e.g., snow, ice), illness (e.g., cold, flu), and lack of transportation. Based upon post-class surveys completed by participants and class leaders, on average 80% of class content was covered per class, all participants were engaged in physical activity, and participants reported that 87% of classes were right on target. In the mid-program evaluation participants reported that they liked exercising and learning together and wanted longer exercise sessions.

Four themes emerged from the focus group, which were motivators, challenges, lifestyle changes, and recommended program changes. Most participants emphasized that exercising and learning about nutrition in a group setting was a large motivator to attend classes each week. One participant reported that “when I’m alone I don’t feel like doing it (exercise) However, when I started coming here, I knew I had to come and I had everything ready to come, and I was motivated to come”. They also reported that they enjoyed making new friends. As one stated, “what I liked is that you learn lots of things from other people, and they help you and understand”. Participants reported that they felt more motivated when their families accepted the recipes that they had learned in the nutrition portion of the class and when they wanted to exercise with them. Initially, participants reported that it was challenging to get family members to support the changes. As one described, “But my challenge was that they learned so that I didn’t have to be struggling; having to cook one food for me and another thing for them”. However, several stated that over time their families came to support exercising and eating healthy meals. One stated, “My family has supported me quite a lot in the sense that they accept the healthy foods; sometimes we go out together for a walk or to play basketball. I would tell them all the things I learned here about working out and why you have to do it”. Another participant noted, “The same happens to me; I learned here; I taught my daughters and my husband”. Continued challenges included portion control, weight fluctuations, lack of time, and lack of family support. As one described, “most of the time I eat healthy now. The only thing I have to remember is not to eat too much, smaller portions”. Many of the women said they continued to use recipes and have maintained and expanded their exercise program after the classes ended. For example, one participant said “I’m using weights. Now my body feels strong. When I went to Walmart I couldn’t lift the 50-pound bags, and now I can lift them up; I don’t have to ask for help”. Recommended program changes included offering the program three days a week, longer exercise sessions, and longer program duration (longer than 12 weeks).

## 4. Discussion

We assessed the efficacy and feasibility of an adapted physical activity and nutrition program for rural Latinas. This pilot study provides preliminary evidence to suggest that *Mujeres Fuertes y Corazones Saludables* can improve weight, fitness, physical activity, and diet in rural Latinas. Participants experienced significant changes in weight, BMI, waist circumference, cardiovascular fitness, consumption of sugar-added drinks and fruits and vegetables, and physical activity self-efficacy. There was a trend toward improvements in dietary behavior and self-efficacy. The changes in weight observed in this pilot study (−1.5 kg, post-pre) are comparable to changes seen in the randomized trial of the original evidence-based physical activity and nutrition program, StrongWomen—Healthy Hearts, which demonstrated a significant decrease in weight (−2.1 kg) compared to a delayed intervention control group [14]. Thus, the adapted program has retained similar efficacy as the original program. Participants reported that the group-based program was an important motivator for attending classes and for making changes in diet and physical activity. We found that it was feasible to hold in a rural community organization serving Latinas.

It is important to emphasize that small lifestyle changes can have an impact. A reduction in sugar-added drinks of 44% and more than half a serving per day is promising since studies have consistently shown a significant association and direct dose–response relationship between sugar-added drinks, long-term weight gain, and risk of type 2 diabetes [32]. Additionally, an increase in physical activity self-efficacy during the active phase of an intervention can be predictive of physical activity and weight loss at later time points [33].

There are other lifestyle behavior change programs aimed at promoting physical activity and/or healthy eating with Latina participants. However, these programs vary in duration (e.g., 12 weeks, one year), intensity (e.g., bi-weekly, monthly), types (e.g., classroom, active exercise), content (physical activity, diet, heart disease), outcomes and measurement of outcomes (minutes/week of physical activity, BMI, fruit and vegetable intake, cardiorespiratory fitness), and target population (urban/rural, female only), making it difficult to compare across studies. For example, in *Pasos Adelante*, which is a 12-week program that includes weekly two-hour classroom sessions covering topics including physical activity, heart disease, diabetes, and diet, as well as walking clubs held for 20 minutes weekly, participants (90% women) residing in rural border communities decreased BMI by 1% and waist circumference by 4% [34]. In *Camina por Salud*, urban obese Mexican American women (ages 45–70) were assigned to walk for 30 minutes three days per week (90 minutes total) or five days per week (150 minutes total) and could participate in a weekly *promotora*-led walking group for 36 weeks [33]. Participants did not achieve target goals of minutes per week in either group; however, they had decreases in BMI (6% and 7%), weight (12% and 17%), and waist-hip-ratio (1% and 1%) at 36 weeks [35]. In *Familias Sanas y Activas*, *promotoras* offered no-cost moderate intensity exercise classes and walking groups throughout the week in selected locations in urban neighborhoods targeting Latinos and also provided a physical activity resource kit to participants [34]. At six months, participants had decreases in waist circumference and systolic blood pressure and increases in fitness and hamstring flexibility but no change in weight, BMI, or diastolic blood pressure (specific pre–post measurements not provided) [36]. Other programs aimed at Latinas have found changes in targeted outcomes but not necessarily all of the targeted outcomes [29,33]. This variance speaks to the different goals, culture, socioeconomics, and resources of Latina communities as well as the individual and environmental factors impacting adoption and maintenance of behavior change. Thus, determining which program will be successful = likely depends upon the local context and the salience of that program to address the determinants and goals of behavior change (exercise and diet) for that particular community.

Despite strong community partnerships and participant interest, both recruitment and retention were challenging within this limited resource population with periods of under- and unemployment. Steps we took to address these challenges included having known and trusted community members recruit participants and lead the class, offering the program during the winter months when many women are not engaged in seasonal farm work, and connecting with participants who missed class to problem solve barriers to attendance. These strategies, albeit resource-intensive, have been employed by other researchers to recruit and retain Latinas in research and prevention programming with success [25,37]. The attrition rate of 26% in this pilot study is similar to the attrition rate in other lifestyle behavior change programs with Latinas [34,35]. Thus, recruiting and retaining rural Latinas, in particular farmworkers (66% participants were farmworkers), in a lifestyle program is a continued challenge that requires sensitivity to the unique challenges experienced by this population including seasonal agricultural work, limited individual resources, dispersed population, changing phone numbers, lack of trust in research and researchers, and fear of deportation, and using resource intensive strategies tailored to the local context to address these challenges.

This pilot study, focused on assessing the feasibility and efficacy of an adapted physical activity and nutrition program within a rural Latina population, has some limitations. The small sample size and one group pre–post design limited our ability to fully measure the effect size and generalizability of the findings. However, a notable strength of this study was that it reached a marginalized and difficult to engage community, rural Latina farmworkers.

## 5. Conclusions

This pilot study of a culturally adapted behavior change program to promote physical activity and healthy eating in middle-aged, overweight, sedentary, rural Latinas showed promising results; however, this was a one-group pre–post pilot study with a small sample; a randomized trial with a larger sample is needed to establish effectiveness prior to dissemination.

## Figures and Tables

**Table 1:** *Mujeres Fuertes y Corazones Saludables* class overview.

Class Session	Physical Activity Content (30 minute)	Nutrition and Behavior Change Content (30 min)
(1) Women and Heart Disease	Aerobic dancing/ aerobics DVD 5-minute cool down	The heart and heart disease
		Signs and symptoms of a heart attack in women
(2) Weight Control for Heart Health, Part 1	Aerobic dancing/ aerobics DVD 5-minute cool down	Specific, Measurable, Achievable, Relevant and Time-bond (S.M.A.R.T).) goals and plans
		Food and exercise logs
		Exercise intensity
		Added sugars and sugary drinks
(3) Weight Control for Heart Health, Part 2	Aerobic dancing/aerobics DVD 5-minute cool down	Choosing type of exercise
		Portion control
		Activity: Portion Distortion Interactive Quiz
(4) Heap on the Vegetables and Fruits, Part 1	Aerobic dancing/aerobics DVD 5-minute cool down	Exercise soreness/discomfort—what to expect
		Getting vegetables and fruit throughout the day Revisiting S.M.A.R.T plans
(5) Heap on the Vegetables and Fruits, Part 2	Aerobic dancing/ aerobics DVD 5-minute cool down	Food preparation activity: Healthy vegetables and salads
(6) Emphasize the Right Fats, Part 1	Aerobic dancing/ aerobics DVD 5-minute cool down	Different types of fat and fats in foods
		Activity: Reading fat content on food labels
		S.M.A.R.T. plans check-in
(7) Emphasize the Right Fats, Part 2	Aerobic dancing/ aerobics DVD 5-minute cool down	Food preparation activity: *Frijoles refritos a la*
		*Veracruzana* and whole wheat tortillas
(8) Accentuate Whole Grains, Part 1	Aerobic dancing/ aerobics DVD 5-minute cool down	Whole grains and portions
		Activity: Reading whole grain info on Nutrition Facts Panels
		S.M.A.R.T. plans check-in
(9) Accentuate Whole Grains, Part 2	Aerobic dancing/ aerobics DVD 5-minute cool down	Tasting and rating whole grains (e.g., bulgur, millet, quinoa) and whole grain salads
(10) Revere Low- and Nonfat Dairy, Part 1	Aerobic dancing/ aerobics DVD 5-minute cool down	Dairy and getting dairy in diet
		Activity: Reading dairy info on Nutrition Facts Panels
		S.M.A.R.T. plans check-in
(11) Revere Low- and Nonfat Dairy, Part 2	Aerobic dancing/ aerobics DVD 5-minute cool down	Food preparation activity: Vegetables and salads—low fat
(12) Target Heart-Healthy Proteins, Part 1	Aerobic dancing/ aerobics DVD 5-minute cool down	Proteins (fish, meats, poultry, eggs) and portions
		S.M.A.R.T. plans check-in
(13) Target Heart-Healthy Proteins, Part 2	Aerobic dancing/ aerobics DVD 5-minute cool down	Food preparation activity: Baked fish and lemon sauce, bean and corn salad, tuna salad
(14) Putting It All Together, Part 1: Grocery Shopping, Breakfast	Aerobic dancing/ aerobics DVD 5-minute cool down	Breakfast
		Grocery shopping and budget
		Activity: Making breakfast choices
		S.M.A.R.T. plans check-in
(15) Putting It All Together, Part 2: Lunch, Salt	Aerobic dancing/ aerobics DVD 5-minute cool down	Review breakfast
		Lunch
		Salt and spices
(16) Putting It All Together, Part 3: Snacks, Nuts, Treats	Aerobic dancing/ aerobics DVD 5-minute cool down	Review lunch
		Snacks and treats
		Role of nuts
		S.M.A.R.T. plans check-in
(17) Putting It All Together, Part 4: Dinner, Healthy Cooking, Alcohol	Aerobic dancing/ aerobics DVD 5-minute cool down	Review snacks and treats
		Dinners
		Strategies for maintaining goals and progress
(18) Putting It All Together, Part 5: Menu Planning	Aerobic dancing/ aerobics DVD 5-minute cool down	Menu planning
		Modifying traditional foods to be healthy and tasty (e.g., *carnitas*)
		S.M.A.R.T. plans check-in
(19) Putting It All Together, Part 6: Menu Planning, Eating By Heart and Weight Loss	Aerobic dancing/ aerobics DVD 5-minute cool down	Menu planning revisited
		Weight loss and healthy eating
		Instructions on completing Home Environment
		Assessment
(20) Weight Control for a Lifetime, Part 1	Aerobic dancing/ aerobics DVD 5-minute cool down	Review completed Home Environment Assessments
		Creating healthy food environment in home and strategies to control eating (e.g., mindful eating)
		S.M.A.R.T. plans check-in
(21) Weight Control for a Lifetime, Part 2	Aerobic dancing/ aerobics DVD 5-minute cool down	Eating in restaurants
		Activity: Role-playing restaurant ordering
(22) Weight Control for a Lifetime, Part 3	Aerobic dancing/ aerobics DVD 5-minute cool down	Family and friends support and sabotage
		Social events and healthy eating
		Activity: Role-playing communicating for weight loss
		S.M.A.R.T. plans check-in
(23) Stress Reduction	Aerobic dancing/ aerobics DVD 5-minute cool down	Stress, hostility, and depression and heart health
		Regaining emotional equilibrium (e.g., stress reduction)
		Meditation exercise
(24) Heart Healthy Potluck and Awards Ceremony		Healthy foods potluck and awards ceremony

**Table 2:** Sociodemographic characteristics of participants at baseline and outcome assessments.

	Baseline Assessment	Outcome Assessment
*n*	15	11
Age (years)	52.2	
Low acculturation	71.4 %	
Farmworker	66%	
	Baseline *n* (%)	Outcome *n* (%)
Employment		
Full time or Part time	4 (26.7)	5 (45.5)
Not employed	10 (66.7)	6 (54.5)
Income		
<U.S $15,000	8 (53.5)	2 (13.3%)
U.S. $15,000–$34,999	2 (13.3)	3 (20.0)
U.S. $35,000–$49,999	2 (13.3)	2 (13.3)
U.S. $50,000–$99,999	1 (6.7)	1 (6.7)
≥U.S. $100,000	0 (0.0)	1 (6.7)
Marital status		
Married, or married-like relationship	10 (66.7)	10 (90.9)
Divorced or separated	2 (13.3)	1 (9.1)
Single	3 (20.0)	0 (0)
Education		
Less than 7th grade	4 (26.7)	4 (36.4)
Less than 11th grade	4 (26.7)	3 (27.3)
High school graduate	3 (20.0)	1 (9 0.1)
Some college	1 (6.7)	1 (9 0.1)
College graduate +	1 (6.7)	1 (9 0.1)

**Table 3:** Changes in anthropometric variables, physical fitness, diet, and self-efficacy.

	Pre-Intervention ^a^ Mean (SD)	Post-Intervention ^a^ Mean (SD)	%Mean Change Post—Pre ^a^	Post—Pre within Group Difference (95% CI)	*p* ^b^	Effect size (Cohen’s D) ^c^
*n*	15	11	−26.7%	-	-	-
Weight (kg)	78.9 (12.6)	77.4 (12.4)	−1.9%	−1.5 (−2.5, −0.5)	**0.009**	0.12
BMI	34.3 (4.2)	33.7 (4.5)	−1.7%	−0.6 (−1.0, −0.2)	**0.005**	0.14
Waist circumference (cm)	102.4 (11.1)	99.4 (11.7)	−3.0%	−3.0 (−5.0, −1.0)	**0.008**	0.26
6-minute walk test (meters)	414.1 (56.2)	483.7 (47.1)	16.8%	69.7 (51.2, 88.1)	**<0.001**	1.34
Diet frequency (servings per day)						
Sugar-added drinks	1.6 (1.3)	0.9 (0.9)	−43.8%	−0.7 (−1.2, −0.2)	**0.008**	0.64
Sugar-added food	0.9 (0.8)	0.5 (0.4)	−44.4%	−0.3 (−0.9, 0.2)	0.190	0.52
Fruit	0.9 (0.7)	1.0 (0.9)	11.1%	0.1 (−0.8, 1.0)	0.800	0.13
Vegetables	1.0 (0.7)	0.9 (0.2)	−10.0%	−0.1 (−0.6, 0.4)	0.759	0.14
Whole Grains	0.7 (0.6)	1.2 (1.0)	71.4%	0.5 (−0.5, 1.4)	0.282	0.55
Food Intake Questionnaire (how many times consumed in one day)						
Sugary drinks yesterday	0.9 (0.9)	0.3 (0.5)	−66.7%	−0.6 (−1.1, 0.0)	0.051	0.75
Sugary drinks typically	0.8 (0.8)	0.4 (0.7)	−50.0%	−0.3 (−0.9, 0.2)	0.195	0.44
Fruits and veggies yesterday	2.3 (0.9)	3.7 (1.7)	60.9%	1.3 (0.1, 2.5)	**0.035**	0.98
Fruits and veggies typically	2.6 (1.4)	3.8 (2.2)	46.2%	1.2 (−0.8, 3.3)	0.209	0.66
Self-efficacy (scale of 1–5 with 1 as “Not at all confident” and 5 as “Completely confident”)						
Diet	3.2 (1.0)	3.9 (0.8)	21.9%	0.7 (−0.1, 1.5)	0.079	0.76
Physical activity	2.4 (1.0)	3.3 (1.3)	37.5%	0.9 (0.2, 1.6)	**0.022**	0.78
Physical Activity						
IPAQ (MET-minutes/week) ^d^	1325.3 (2214.7)	1148.0 (1051.2)	−13.4%	−178.3 (−2514.3, 2157.8)	0.824	0.10

^a^ Mean and SD values are reported from paired sample statistics. ^b^ Paired *t*-test used 95% confidence. ^c^ Cohen’s D is presented in absolute value. ^d^ MET is a metabolic equivalent, which is used in order to represent exercise activity. BMI: body mass index. IPAQ: International Physical Activity Questionnaire.
